# Biomarkers in Cystic Fibrosis Lung Disease – A Review

**DOI:** 10.2478/rjaic-2020-0011

**Published:** 2020-10-11

**Authors:** Ioana Mihaiela Ciuca, Paula Marian, Marc Monica

**Affiliations:** 1 Pediatric Department, University of Medicine and Pharmacy “Victor Babes” Timisoara, Timisoara Romania; 2 Department of Medical Disciplines , Oradea University, Faculty of Medicine and Pharmacy, Oradea, Romania; 3 Pulmonology Department, University of Medicine and Pharmacy “Victor Babes” Timisoara, Timisoara Romania

**Keywords:** Cystic fibrosis lung disease, children, biomarkers

## Abstract

Cystic fibrosis is a polymorphic disease, marked by multiple and difficult-to-treat respiratory exacerbations with severe evolution. The lung disease dictates the disease’s evolution and it must be diagnosed early and treated accordingly, but the diagnosis is sometimes challenging because of the lack of a sensible tool. In the era of the biomarkers, the need for a sensitive and reliable one would be extremely important, considering that inflammation secondary to infections produce irreversible structural changes in the cystic fibrosis lungs. The present paper reviews the studied biomarkers in inflammation and infection with potential role in cystic fibrosis lung disease.

## Introduction

From relatively benign conditions like acute respiratory infections to severe diseases, the need for specific biomarkers increases. [1] Cystic fibrosis is a disease with various manifestations, from lung disease to liver disease, with different evolution and limited response to available therapies.[2] Biomarkers are quantifiable indicators, which could define the relation with a disease or a physiological status, being capable of supporting the diagnostic or a predictive assessment of a certain pathology. Accessibility, sensitivity, and specificity are significant factors for biomarker, to be reliable.[3] Pulmonary biomarkers are being used more frequently to monitor malady activity and evaluate response to treatment in individuals with cystic fibrosis (CF). An important direction of biomarkers’ development would be the early detection of pulmonary infections besides efficiency treatment following the detection and disease’s evolution monitoring. With increasing importance of the development of contender anti-inflammatory drugs for cystic fibrosis, the need for identification of reliable biomarkers indicating the effect of the therapy with subsequently showing clinical improvement.[4] If for severe, frequent pathologies like sepsis, a special interest was addressed, for rare diseases like cystic fibrosis, the studies are at their infancy. There are many biomarkers used for inflammation and in the diagnosis and evaluation of severe diseases like sepsis,[5] interleukin 1 (IL-1), interleukin 2 (IL-2), interleukin 6 (IL- 6), interleukin 12 (IL-12), interleukin 8 (IL-8), interleukin 4 (IL-4), interleukin 10 (IL-10), interleukin 17 (IL-17), interleukin 13 (IL-13), tumor necrosis factor alpha (TNF-alpha), besides interferon gamma (INF-gamma), transforming growth factor beta (TGF-beta), procalcitonin (PCT), N-terminal C natriuretic peptide (NT-CNP), C-reactive proteins (CRP), granulocytes and monocytes colony stimulating factor (GMCSF), leukotrienes, prostaglandins and thromboxane, fractions of the complement (C3a, C5a).[6]

Quon published an extensive review of the literature, quantifying the circulating proteins relied to the inflammatory pathways in four directions: acute phase proteins; pro-inflammatory cytokines; markers of neutrophilic inflammation; and markers of tissue injury/hypoxia. Acute phase proteins included C-reactive protein (CRP) and soluble cluster of differentiation 14 (sCD14), for the early phase inflammatory response interleukin-6 (IL-6), interleukin-1β (IL-1β), and monocyte chemoattractant protein-1 (MCP-1) were evaluated. As for neutrophilic inflammation, the markers used were granulocyte colony-stimulating factor (G-CSF) and myeloperoxidase (MPO), vascular endothelial growth factor (VEGF) being considered as a marker of tissue injury/hypoxia.[7] The results revealed that sCD14 levels were significantly higher in patients with exacerbation in short-term follow-up, with an ascending trend of significant increase in CRP, MCP-1, and MPO levels. None of the other plasma biomarkers

demonstrated adequate differential capacity for the detection of CF pulmonary exacerbations,[1] although there are studies suggesting other sensitive markers, like blood leucocyte gene expression.[7] For the life expectancy of the patients with cystic fibrosis, the most important fact is to maintain their lungs in a good functional state, despite frequent inflammatory exacerbations secondary to Pseudomonas aeruginosa or other CF specifics microorganism.[8] As a result of inflammatory process, the deterioration of the lung structure, with the development of bronchiectasis and parenchymal destruction lead to a decrease of the lung function,[9] followed by chronic hypoxia and eventually death. The identification of new biomarkers is crucial for an improved CF management of patients with cystic fibrosis. Researchers like Rao found the bacterial miR-146, which binds to a receptor of the TLR family, in the sputum of CF patients infected with *Pseudomonas aeruginosa.*[10]

Another miRNAs, miR-155 seems to activate the IL-8-dependent inflammation in patients with CF; high levels of miR-155 in sterile CF cells decreased after exposure to the anti-inflammatory cytokine IL-10 or following inhibition of IL-1β signaling,[11] followed by decreasing levels of IL-8 production. This advocate that miR-155 is an IL-8 expression regulator and consequently of the NF-κB pathway.[12]

## Infections biomarkers

Patients with cystic fibrosis are at risk of developing chronic pulmonary infection with redoubtable germs; the most dangerous being *Pseudomonas aeruginosa*, with a methicillin-resistant *Staphyloccocus aureus* and *Burholderia spp.*^[8,9]^ *Pseudomonas aeruginosa* colonizes the cystic fibrosis lungs despite aggressive antibiotic treatment; chronic colonization leads to progressive lung damage, by forming bronchiectasis, frequents exacerbations, and finally, respiratory failure and decease in most CF patients. *Pseudomonas aeruginosa* has the ability to develop protective biofilms that protect the microbe against the antibiotic action.

It is well-known that one of the mechanisms that triggers the inflammatory response is the effect of bacterial toxins.[12] Microbes have different ways to fight with the human body, one is discharging one toxin, or more bacterial toxins that produce infection, while others exotoxins with a direct role in toxic shock. There are bacteria producing both exotoxins and endotoxins, while several producing only endotoxins. In the effort of finding a reliable biomarker for the early revealing of the first contact of the cystic fibrosis lung with Pseudomonas aeruginosa, many serological or exhaled biomarkers, were considered by different researchers.

Leeds criteria are currently used for the classification of *P. aeruginosa* infection status; first infection is defined by the first detection of the microbe in the culture, intermittent infection by the presence of intermittent positive cultures for one year and chronic infection as persistence of the positivity of *P. aeruginosa* in the cultures.[13] The utility of serological tests for detection of *P. aeruginosa* pulmonary infection in cystic fibrosis (CF) is debatable. Various reports suggest that serological tests may help in the distinction between intermittent colonization and chronic infection.[14] Some studies sustained the value of the anti-*Pseudomonas aeruginosa* IgG for sensing the infection, concluding that dosage of anti-*P. aeruginosa* IgG can be a valuable tool for the identification of *Pseudomonas aeruginosa* chronic infection in patients with CF. In the last three decades, several studies have shown the potential of antibody detection for early diagnosis of *Pseudomonas aeruginosa* pulmonary infection,[15] in order to initiate the early eradication therapy.

Noninvasive markers were pursued, like fraction of expired nitric oxide (FeNO); FeNO values in patients with cystic fibrosis are known to be lower than FeNO values found in normal subjects. Many factors could contribute to low airway nitric oxide (NO) levels in CF patients and Grasemman showed that allele assignment at an intronic repeat polymorphism in the NOS1 gene is associated with exhaled NO levels in patients with CF.[16] Gaseous hydrogen cyanide (HCN) is present in mouth-exhaled breath of patients with CF, suggesting the presence of *Pseudomonas aeruginosa* in the lower airways, was found in vitro, and confirmed by some studies, with microbiological cultures.[17] There were studies supporting that exhaled breath HCN measurements could be an additional diagnostic tool to detect the early presence of PA in the lower airways and a non-invasive monitor to enhance the likelihood of its eradication. HCN was found in elevated levels in the exhaled air of children with cystic fibrosis compared to children with asthma,[18] but its lack of sensitivity failed in its sustenance as a consistent biomarker for early CF Pseudomonas infection; exhaled hydrogen cyanide being specific, but insensitive biomarker of new *Pseudomonas aeruginosa* infection in children with **CF.**[19] Infections generated by *Pseudomonas aeruginosa* alter the expression of miRNAs, especially miRNA-302b[20] and miRNA-233.[21]

## Conclusions

Although various biomarkers are consistently relied with different aspect of such an exceptional pathology like cystic fibrosis and many of them are promising, more studies are needed for finding the suitable markers. The aim would be not only inflammation but finding of biomarkers for disease’s progress, treatment efficiency and early infections detection. The forthcoming for all of this appears to be the biomarkers.
